# Evaluation of inflammatory hyperreflective foci and plasma EPA as diagnostic and predictive markers for age-related macular degeneration

**DOI:** 10.3389/fnins.2024.1401101

**Published:** 2024-10-10

**Authors:** Zhongqi Wan, Yan Wu, Tianyi Shen, Chengyu Hu, Ruoyi Lin, Chengda Ren, Donghui Yu, Tingting Li, Meijiang Zhu, Wenting Cai, Jing Yu

**Affiliations:** ^1^Department of Ophthalmology, Shanghai Tenth People's Hospital, School of Medicine, Tongji University, Shanghai, China; ^2^Department of Ophthalmology, Renji Hospital, School of Medicine, Shanghai Jiao Tong University, Shanghai, China; ^3^Department of Ophthalmology, West China Hospital, Sichuan University, Chengdu, Sichuan, China; ^4^Department of Ophthalmology, Shanghai First People's Hospital, Shanghai Jiaotong University, Shanghai, China; ^5^Department of Ophthalmology, Shanghai East Hospital, Tongji University School of Medicine, Shanghai, China; ^6^Department of Ophthalmology, The Third People's Hospital of Bengbu, Bengbu, China

**Keywords:** age-related macular degeneration, inflammation, biomarker, polyunsaturated fatty acids, omega-3 fatty acid, eicosapentaenoic acid, hyperreflective foci

## Abstract

**Objectives:**

To detect the plasma polyunsaturated fatty acids (PUFAs) concentrations in age-related macular degeneration (AMD) patients and healthy controls. Additionally, advanced studies were conducted to investigate the relationship between PUFAs concentrations and ophthalmological characteristics, including hyperreflective foci (HRF), visual acuity, and anti-vascular endothelial growth factor (anti-VEGF) response in patients with AMD.

**Methods:**

This prospective, single-site study recruited a total of 315 participants, consisting of 105 individuals with dry AMD (early-stage AMD group), 105 individuals with neovascular AMD (late-stage AMD group), and 105 elderly individuals without any fundus diseases (healthy controls). The levels of omega-3 and omega-6 PUFAs in plasma were detected using gas chromatography. Retinal thickness, choroidal thickness, and macular volume were quantified using optical coherence tomography angiography (OCTA) scan with a 6 × 6 mm macular area, and the amounts of HRF were analyzed with OCTA scanning data.

**Results:**

Compared to the control group, AMD patients exhibited significantly lower plasma concentrations of eicosapentaenoic acid (EPA), docosahexaenoic acid (DHA) and alpha linolenic acid. HRF were observed in various retinal layers of AMD patients, particularly those with late-stage AMD. The correlation coefficient matrix and multiple linear regression models demonstrated that HRF played a crucial role in best corrected visual acuity for both early (*p* < 0.001) and late-stage AMD patients (*p* = 0.006), while EPA had an inverse effect on the logarithm of the minimum angle of resolution (logMAR) value in patients with early-stage AMD (*p* < 0.001). As compared to patients with good responses to anti-VEGF therapy, those with poor responses had significantly lower baseline logMAR (*p* < 0.001), central retina thickness (*p* = 0.002), macular volume (*p* = 0.027), HRF (*p* = 0.024), and plasma EPA (*p* < 0.001). This study used a ROC curve analysis to identify the combination of HRF and EPA as a potential biomarker for predicting the response to anti-VEGF treatment in late-stage AMD patients, with an area under the curve (AUC) value of 0.775.

**Conclusions:**

Reduced plasma EPA was detected in AMD cases and the lower EPA concentration was related to poorer visual acuity. Additionally, the quantity of HRF combined with concentration of plasma EPA may serve as the prognostic indicator for predicting the effect of anti-VEGF treatment in late-stage AMD patients.

## Introduction

Age-related macular degeneration (AMD) is the leading cause of severe vision loss in the elderly all over the world. The prevalence of the disease has increased for several decades and it is expected that 288 million people worldwide will suffer from AMD by 2040 (Wong et al., 2014). According to the clinical manifestations and fundus pathological changes, this disease can be divided into early, intermediate and late AMD. The main feature of early and intermediate AMD is the formation and aggregation of drusen, while late AMD can be neovascular (also called exudative or wet AMD) or atrophic (also called non-exudative or dry AMD) (Fleckenstein et al., 2018; Spaide et al., 2020). The diagnosis and treatment of AMD is still a not only prominent but also challenging issue within clinical practice. Although a variety of anti-vascular endothelial growth factor (anti-VEGF) drugs have been used to control choroidal neovascularization (CNV) in wet AMD, there are still deficiencies in the diagnosis and treatment of AMD (Gross et al., 2018; Zhu et al., 2021). On the one hand, there is a lack of good intervention measures for early and intermediate AMD before progressing to the late AMD. On the other hand, intravitreal injection of anti-VEGF drugs has a series of potential risks, including the invasiveness of intravitreal injection, the potential risk of infection, and the acceleration of the development of geometric atrophy (GA).

At present, the diagnostic and monitoring methods of AMD rely on the observation of fundus structure and visual function. Although both of them are closely related to the severity of the patient's disease, they are still limited in the early diagnosis of the disease, the prediction of disease progression and the judgment of the efficacy of anti-VEGF therapy (Ly et al., 2018). Considering the technology and cost, early screening and detection methods inevitably need to be developed in the direction of being low cost and non-invasive. Given this, circulating markers and non-invasive ocular examination methods have natural advantages. At present, inflammatory factors (Mimura et al., 2019), antioxidant factors (Chew et al., 2013) and single nucleotide polymorphism (SNP) mutation situations (Hagstrom et al., 2015) have been used as potential biomarkers of AMD. In addition, the value of imaging examination in the diagnosis and treatment of AMD has also been clarified in a number of studies, which is crucial in the early diagnosis of the disease, subtype judgment and prognosis analysis (Schmidt-Erfurth and Waldstein, 2016; Sadda et al., 2018; Fragiotta et al., 2018). Though, many relevant studies have been reported, there are few studies that combine circulatory system markers and tissue structure information.

Carboxyl groups with hydrocarbon chains make up fatty acids, which can be saturated or unsaturated. Fatty acids function diversely, and are categorized based on the number of carbon atoms and double bonds they possess. Saturated fatty acids (SFAs) have no double bonds. Monounsaturated fatty acids (MUFAs) contain a single double bond, while polyunsaturated fatty acids (PUFAs) contain two or more. PUFAs are composed of omega-3 fatty acids and omega-6 fatty acids, depending on the location of their initial double bond. Metabolites derived from omega-3, specifically eicosapentaenoic acid (EPA), and docosahexaenoic acid (DHA), have been reported to possess anti-inflammatory and anti-angiogenic properties, while those derived from omega-6 exhibit contrasting effects (Heesterbeek et al., 2020). Many studies have found that the anti-inflammatory properties of omega-3 PUFAs (Merle et al., 2013, 2011; Christen et al., 2011) have a protective effect on AMD since inflammation appears to have a pivotal role in this disease (Donoso et al., 2006). It was also reported by several longitudinal studies that there was a decreased risk of developing AMD, consuming higher amounts of DHA or EPA (Reynolds et al., 2013; Ho et al., 2011; Chiu et al., 2009). Another study demonstrated that the usage of PUFAs for nutritional support in the treatment of AMD can reduce the need for anti-VEGF drugs (Semeraro et al., 2019). Therefore, it is vital to investigate the exact impact of PUFAs on the incidence, progression, or treatment response of AMD through well-designed studies.

Previous researches (Brito et al., 2024; Merle et al., 2014; Lawrenson and Evans, 2012) have reported lower levels of omega-3 PUFAs or a higher ratio of omega-6 to omega-3 PUFAs in AMD patients, yet the relationship between PUFAs levels and ophthalmological characteristics in AMD cases remains uncertain.

The advancement of optical coherence tomography (OCT) technology has facilitated the enhanced visualization of the interlamellar structure of the retina. Additionally, the emergence of scattered spotted reflection signals in the retina has been progressively observed. Hyperreflective foci (HRF) was first described by Bolz et al. (2009). In patients with diabetic macular edema (DME), Matthias et al. had observed HRF located around the vascular wall of retinal microaneurysms and dispersed throughout all layers of the retina. It is postulated that these HRF may represent lipoprotein exudated resulting from the disruption of the blood-retinal barrier, or can be considered as precursors to hard exudation. Subsequently, as ophthalmologists have conducted extensive investigations into retinal high reflex points and continuously improved OCT equipment, our comprehension of these phenomena has been consistently updated. The characteristics typically associated with HRF on OCT include a diameter ranging from 20 to 40 μm, signal intensity comparable to that of the retinal pigment epithelium, a dotted and discrete distribution, a distinct boundary, and a prevalence throughout the various layers of the retina, primarily concentrated in the outer layer (Nassisi et al., 2018; Christenbury et al., 2013). However, the origin of the elevated reflex point of the retina remains a subject of ongoing debate, with numerous hypotheses proposed.

This study is a prospective clinical study designed to evaluate the diagnostic and predictive value of PUFAs and HRF in patients with AMD. The specific objectives of this study are to investigate the abnormal concentrations of PUFAs in the plasma of AMD patients and healthy controls and also to evaluate the relationship between key PUFAs and visual acuity in AMD cases in Chinese elderly population. Moreover, a short-term follow-up study was conducted to explore whether a type of PUFAs and HRF could be combined biomarkers to predict the effect of anti-VEGF therapy.

## Materials and methods

### Standard protocol approvals

This prospective, single-site study received approval from the Ethical Committee of Shanghai Tenth People's Hospital and was registered in the China Clinical Trials Registration Center (ChiCTR2100051795). The study was conducted in the Department of Ophthalmology of Shanghai Tenth People's Hospital, which is affiliated with the School of Medicine of Tongji University, in accordance with the principles outlined in the Declaration of Helsinki. Prior to enrollment, all participants or their legal guardians provided written consent.

### Study design

**Figure 1:**
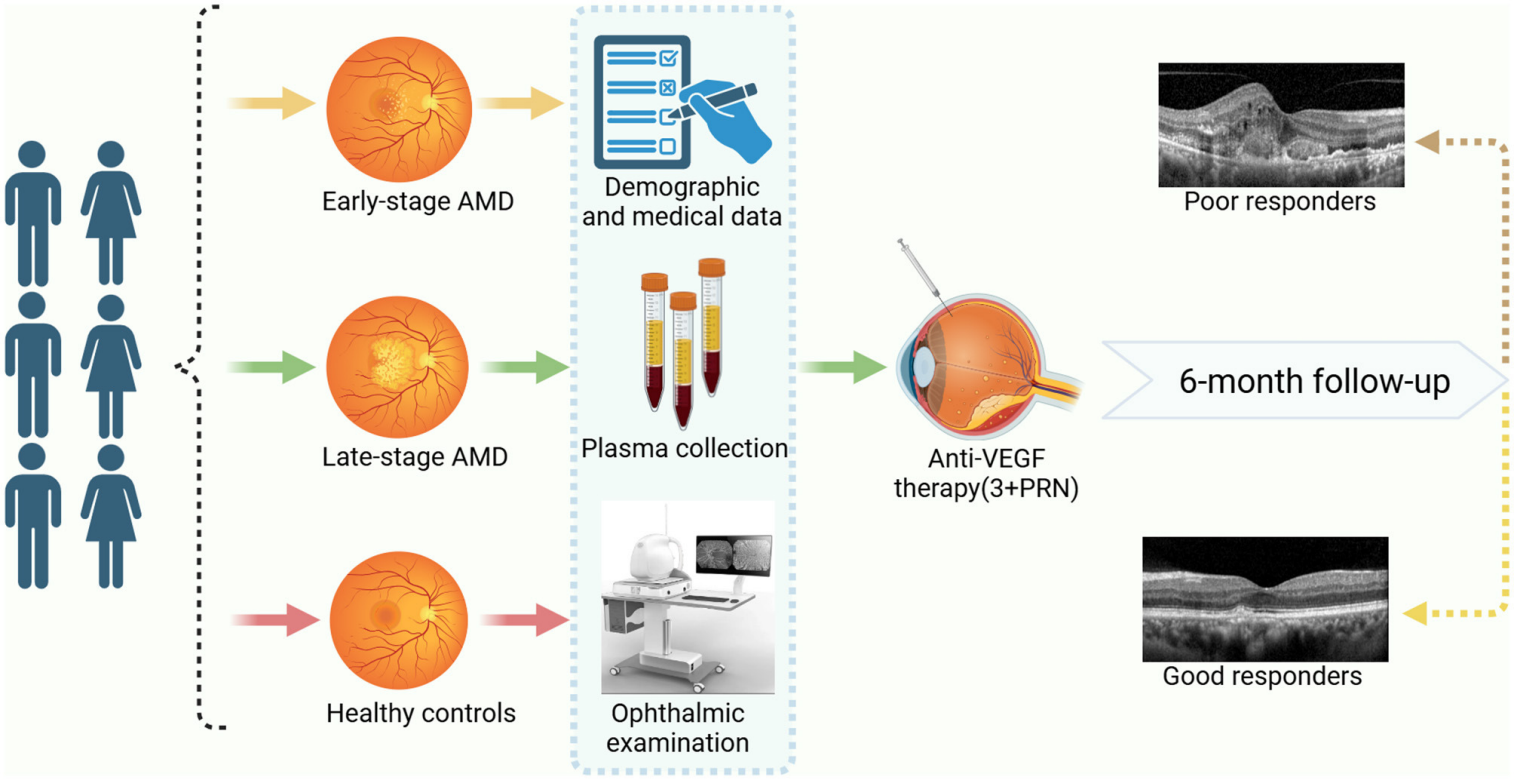
The study workflow. Created with BioRender.com.

This study included a sample size of 315 individuals, comprising three groups: the early-stage AMD group (*n* = 105), the late-stage AMD group (*n* = 105), and the healthy controls (*n* = 105). All participants underwent a comprehensive ophthalmic examination, which encompassed intraocular pressure examination, visual acuity test using the logarithm of the minimum angle of resolution (logMAR) chart, optometry, slit lamp biomicroscope examination, digital fundus photography, and optical coherence tomography angiography (OCTA). Furthermore, the diagnosis of all patients with neovascular age-related macular degeneration (nAMD) was conducted by retinal expert and confirmed through the use of fluorescein angiography (FFA) and indocyanine green angiography (ICGA). Additionally, at the time of enrollment, comprehensive socio-demographic information and medical history records were gathered for all participants, encompassing variables such as age, gender, smoking and drinking habits, body mass index (BMI), physical activity levels, self-reported incidences of hypertension, diabetes, cardiovascular diseases, and hyperlipidemia. Ever smokers were those who smoked at least one cigarette per day for over 3 months and ever alcohol drinking was identified as consuming any alcoholic beverages at least once per week for over 6 months (Zeinomar et al., 2019). Moreover, subsequent to enrollment, blood samples were obtained from all subjects for the purpose of biochemical analysis and capillary gas-liquid chromatography was used to detect PUFAs (Figure 1).

### Inclusion and exclusion criteria

All participants underwent a standardized and thorough ocular examination and were willing to adhere to scheduled follow-ups and provide fasting blood samples. Patients diagnosed with AMD were categorized into either the early-stage or late-stage AMD group based on their pathological alterations. All AMD patients did not take any PUFAs supplements within 1 year before enrollment and did not receive any treatment related to AMD.

Additionally, it was necessary to exclude individuals with high myopia, glaucoma, a history of uveitis, media opacity caused by cataract or corneal diseases, vitreous hemorrhage, epimacular membrane, macular hole, retinal detachment, retinal artery occlusion, retinal vein occlusion, diabetic retinopathy, optic neuropathy, and any other eye diseases that might impact vision, as well as those who had undergone vitrectomy or laser treatment. The inclusion criteria for the early-stage AMD group encompassed individuals with medium-sized drusen (63–125 um), and the absence of evidence of late AMD signs, such as neovascularization and/or geographic atrophy. Patients with macular neovascularization solely associated with AMD determined by OCTA, FFA, and ICGA would be included in the late-stage AMD group. In cases where patients exhibited significant differences in fundus presentations between their two eyes, the more severely affected eye was selected for analysis. The normal control group consisted of elderly volunteers who were matched in age with the AMD group. It was ascertained through a comprehensive ophthalmic examination that they did not exhibit any fundus diseases.

Exclusion criteria encompassed individuals who had undergone intravitreal drug injection therapy, those with rheumatism, immune system disorders, blood system disorders, tumors, genetic diseases, and subjects with poor image quality due to excessive eye movements, and other subjects that researchers thought should be excluded.

### Sample collection and PUFAs measurement

After enrollment, fasting blood samples were obtained from a total of 315 participants and subsequently conveyed to the laboratory for analysis. The determination of plasma concentrations of omega-3 and omega-6 PUFAs, including ALA (C18:3ω3), EPA (C20:5ω3), DHA (C22:6ω3), DPA (C22:5ω3), AA (C20:4ω6), DGLA (C20:3ω6), ADA (C22:4ω6), GLA (C18:3ω6), DPA (C22:5ω6), and LA (C18:2ω6), were performed using gas chromatography on the blood samples, following the guidelines provided by the manufacturer. A volume of 0.5 ml of plasma was taken and placed in a test tube. Subsequently, 2 ml of anhydrous methanol-benzene-sodium hydroxide lye (CH30H-C6H6-NaOH,30:20:1) was added and the mixture was allowed to stand for a duration of 20 min. Following this, 1 ml of 0.5 mol/L methanol-hydrochloric acid and 1 ml of cyclohexane were added and the resulting solution was vigorously shaken and subsequently centrifuged (3,000 r/min). The cyclohexane layer was then extracted for the purpose of conducting combined analysis using gas chromatography/mass spectrometry (GC/MS). The gas chromatography (cc) conditions employed were as follows: a DB-23 chromatographic column was utilized, with a programmed temperature of 60°C for 1 min, followed by a gradual increase to 200°C at a rate of 20°C per minute. This temperature was maintained for a duration of 18 min. The temperature of the injection chamber was set at 200°C, the split ratio was 5:1, and the injection volume was 0.5 microliter. The mass spectrometry (MS) conditions utilized in this study included an ion source temperature of 180°C, an ionization mode of EI source at 70eV, a scanning range of 30 to 500 amu, a delay time of 3 min, and an interface temperature of 180°C. The technicians responsible for conducting the analysis were unaware of the clinical data, ensuring blinding.

### Laboratory measurements

Routine blood tests were conducted by an automatic biochemical analyzer on all individuals, encompassing the detection of lymphocytes, monocytes, neutrophils, C-reactive protein (CRP), cholesterol, triglyceride (TG), Creatinine (Cr), and blood urea nitrogen (BUN).

### OCTA examination and HRF measurements

OCTA (Optovue RTVue XR Avanti, Optovue, Inc.) was performed to scan macular images of the patients, encompassing a 6 × 6 mm area centered on the fovea. Central retinal thickness and choroidal thickness were measured using automated segmentation software, which calculates the average thickness over the scanned area. Manual refinement was applied where necessary to ensure accurate segmentation. HRF counts were performed across the entire macular area within the same predefined region. The HRF count was conducted through manual enumeration by two proficient physicians. To ensure accuracy and mitigate the influence of extraneous signals, the maximum diameter of HRF was limited to a range of 20 μm to 50 μm, thereby excluding insignificant counting noise and preventing the inclusion of prominent hyperreflective clusters, which were typically identified as characteristic hard exudates in fundus photography. Additionally, images of inadequate quality, as indicated by a signal strength index below 7/10, were excluded from the analysis.

### Follow-up and categorized the response to anti-VEGF

All late-stage AMD patients enrolled in the study would be administered intravitreal anti-VEGF drug injections and undergo a continuous regimen of 3+pro re nata (PRN) with monthly assessments. Individual characteristics were likely to contribute to heterogeneity in anti-VEGF drug responses. Morphological and functional outcomes resulting from anti-VEGF treatments would be utilized to assess the therapeutic response after 6 months of enrollment in late-stage AMD patients.

A good response was defined as the complete resolution of intraretinal fluid (IRF) and sub-retinal fluid (SRF), as well as an improvement in best corrected visual acuity (BCVA) of 0.1 logMAR or greater. Patients who exhibited less than 25% reduction in CRT from the initial measurement, along with persistent or increased IRF, SRF, and/or less than 0.1 logMAR improvement in visual acuity compared to the baseline, and experienced further deterioration in vision despite receiving standardized anti-VEGF therapies, were classified as poor responders.

### Statistical analysis

The statistical analysis was conducted using SPSS 22.0 software, while the generation of graphs was facilitated by GraphPad Prism software, version 8.00 (GraphPad Software, La Jolla, CA). All values were reported as either a numerical value or as the mean ± standard deviation (SD). The visual acuity was expressed as the logMAR. Student's *t* test and Pearson chi-square test were employed to compare the information of socio-demographic, lifestyle, laboratory examinations, ophthalmological characteristics, and omega-3 and omega-6 PUFAs concentrations between the early/late-stage AMD group and healthy controls. Correlations between quantitative variables (such as HRF or plasma EPA) were assessed by Pearson correlation coefficient. Multiple linear regressions were utilized to identify independent influencing factors for BVCA in both early and late-stage AMD cases, with *p-*values <0.05 indicating statistical significance. The receiver operating characteristic (ROC) curve and area under the curve (AUC) were used to analyze the diagnostic performance.

## Results

### Patients characteristics

Among the 315 participants, there were no disparities in age, sex, lifestyle or medical histories between early-stage AMD patients and controls except for slight difference in physical activity and self-declared hypertension. Meanwhile, late-stage AMD patients exhibited a higher prevalence of smoking (*p* = 0.043), drinking (*p* = 0.044), and self-reported diabetes (*p* = 0.037) compared to healthy controls. The levels of neutrophils (*P* = 0.034) and CRP (*P* < 0.001), indicative of inflammation, were higher in AMD patients compared to healthy controls based on blood test results.

In this current study, as for ophthalmological characteristics, there was no significant difference between AMD patients and healthy individuals in terms of spherical equivalent and intraocular pressure (IOP). Meanwhile, both early-stage and late-stage AMD patients had reduced choroidal thickness (*p* < 0.001) and thicker central retina (*p* < 0.001) compared to the healthy controls. The results also showed that there was an increase in macular volume (*p* < 0.001), HRF (*p* < 0.001), and logMAR (*p* < 0.001) for two groups of AMD patients compared with the control group respectively (Table 1).

**Table 1:** Socio-demographic, life style, blood tests, and ophthalmological characteristics in early/late-stage AMD and healthy controls.

**Category**	**Characteristics**	**Healthy controls (*n =* 105)**	**Early-stage AMD cases (*n =* 105)**	**Late-stage AMD cases (*n =* 105)**	***p*-value^a^**	***p*-value^b^**
Socio-demography	Age (years)	69.71 ± 8.48	67.46 ± 8.36	69.55 ± 7.53	0.055	0.239
		Gender (male/female)	58/47	52/53	56/49	0.490	0.890
Life styles	Smoking history (ever/never)	30/75	43/62	45/60	0.082	0.043
		Alcohol drinking (ever/never)	9/96	16/89	20/85	0.200	0.044
		BMI	23.92 ± 3.24	23.05 ± 4.13	23.96 ± 3.01	0.092	0.962
		Physical activity				0.049	0.059
		None or casual	53	69	66				
		Frequent moderate	42	32	36				
		Frequent heavy	10	4	3				
Medical histories	Hypertension (Yes/No)	27/78	45/60	39/66	0.013	0.102
		Diabetes (Yes/No)	19/86	23/82	33/72	0.605	0.037
		Cardiovascular diseases (Yes/No)	23/82	32/73	23/82	0.209	1.000
		Hyperlipidemia (Yes/No)	26/79	36/69	27/78	0.173	1.000
Blood tests	Lymphocytes (10^9^/mL)	1.70 ± 0.61	1.69 ± 0.54	1.67 ± 0.53	0.882	0.663
		Monocytes (10^9^/mL)	0.39 ± 0.09	0.39 ± 0.09	0.40 ± 0.11	0.895	0.503
		Neutrophils (10^9^/mL)	3.81 ± 0.70	4.15 ± 1.60	4.20 ± 1.59	0.047	0.034
		Neutrophils-to-lymphocytes	2.58 ± 1.18	2.85 ± 1.78	3.07 ± 3.19	0.203	0.156
		Monocytes-to-lymphocytes	0.27 ± 0.13	0.27 ± 0.19	0.29 ± 0.33	0.703	0.479
		CRP (mg/L)	1.58 ± 1.10	2.35 ± 1.52	2.30 ± 1.44	<0.001	<0.001
		TG (mg/d)	144.57 ± 47.58	148.02 ± 64.51	146.70 ± 53.16	0.661	0.723
		Cholesterol (mg/dL)	195.01 ± 31.86	188.39 ± 36.81	194.40 ± 32.50	0.167	0.946
	BUN (mg/dL)	15.31 ± 2.67	15.04 ± 2.09	15.98 ± 2.38	0.418	0.067
	Cr (mg/dL)	0.83 ± 0.11	0.85 ± 0.11	0.86 ± 0.14	0.407	0.139
	Spherical equivalent (D)	−0.29 ± 1.77	−0.37 ± 1.51	−0.56 ± 1.10	0.734	0.203
Ophthalmological	IOP (mmHg)	16.04 ± 3.13	15.78 ± 3.82	16.74 ± 3.37	0.596	0.108
		logMAR	0.16 ± 0.12	0.31 ± 0.23	0.50 ± 0.16	<0.001	<0.001
	characteristics	Central retinal thickness (μm)	240.71 ± 22.57	260.29 ± 34.35	399.37 ± 123.99	<0.001	<0.001
		Choroidal thickness (μm)	234.24 ± 46.80	178.19 ± 43.32	146.87 ± 42.28	<0.001	<0.001
	Macular volume (mm^3^)	10.31 ± 0.33	11.28 ± 1.00	13.32 ± 1.68	<0.001	<0.001
	HRF	0.04 ± 0.19	7.51 ± 6.42	14.47 ± 9.04	<0.001	<0.001

^a^Mean healthy controls vs. early-stage AMD cases; ^b^mean healthy controls vs. late-stage AMD cases. *p*-value <0.05 indicating statistical significance. BMI, body mass index; CRP, C-reactive protein; TG, triglyceride; BUN, blood urea nitrogen; Cr, Creatinine; D, diopter; IOP, intraocular pressure; logMAR, logarithm of the minimum angle of resolution; HRF, hyperreflective foci.

**Figure 2:**
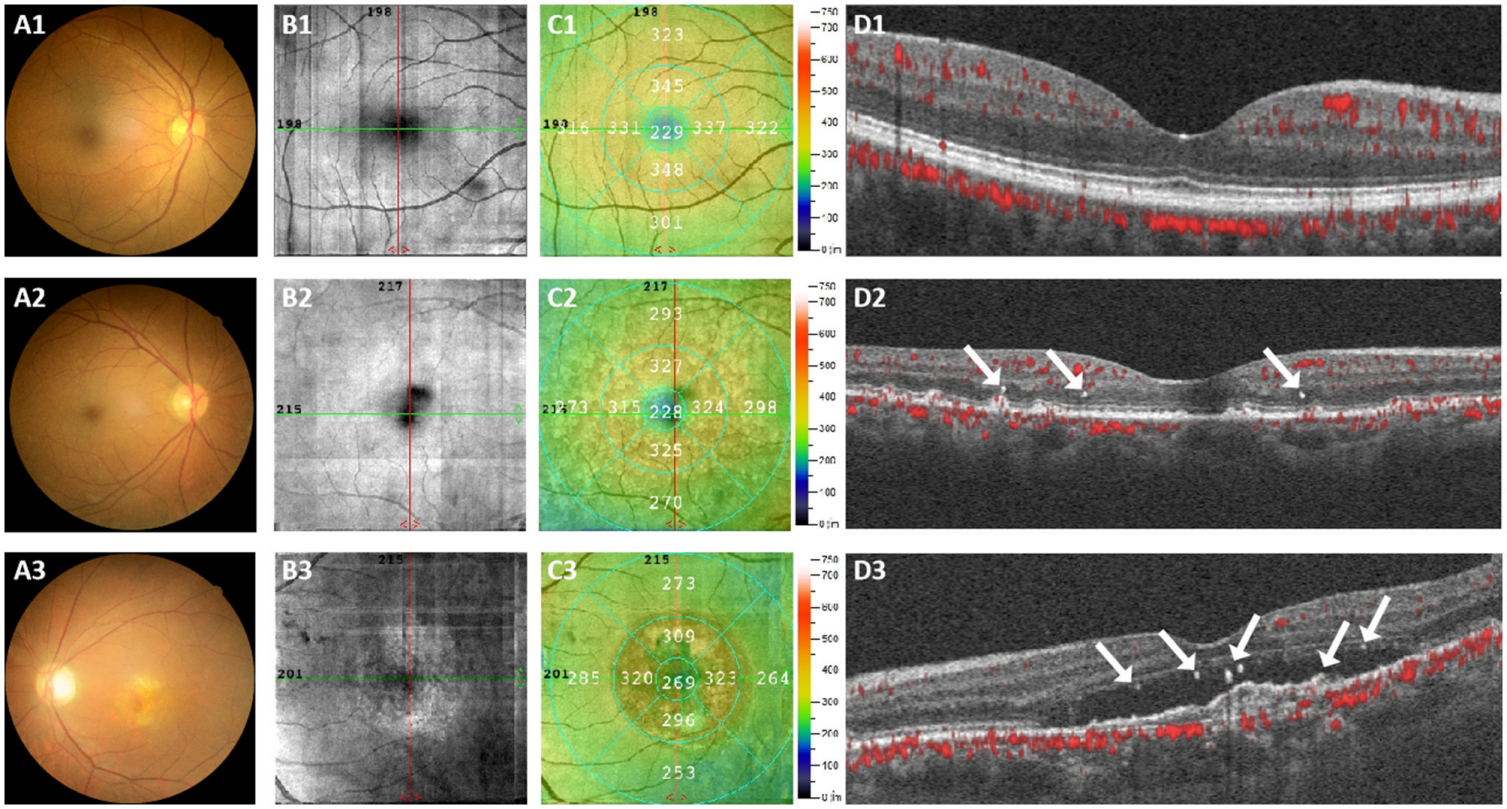
Representative color fundus photography and OCTA images were obtained from three subjects. **(A1–D1)** Represents a normal individual, **(A2–D2)** represents an early-stage AMD patient, and **(A3–D3)** represents a late-stage AMD patient. The presence of the HRF was indicated by a white arrowhead.

The ophthalmological characteristics were analyzed in different groups and Figure 2 displayed the fundus photography and OCTA images of three subjects, representing the healthy controls group, early-stage AMD group and late-stage AMD group respectively. The fundus photography and OCTA image of the healthy individual exhibited a normal fundus structure (Figures 2A1–D1). Conversely, Figures 2A2–D2 illustrated early pathological features of retinal degenerations, such as drusen and disorganization of the normal retinal structure in the images of the early-stage AMD patient. Additionally, the OCTA images revealed the presence of HRF in the deep capillary plexus (DCP) and outer retina (Figure 2D2). Various retinal microstructure lesions, such as choroidal neovascularization, SRF accumulation, increased retinal thickness, and disruptions in the outer limiting membrane, ellipsoid zone, and interdigital zone/retinal pigment epithelial layer, were observed in the images of the late-stage AMD patient (Figures 2A3–D3). Figure 2D3 depicted the distribution of HRF across all retinal layers, encompassing the superficial capillary plexus (SCP), DCP, and outer retina. The HRF were indicated by a white arrowhead.

### The PUFAs concentrations in AMD cases and healthy controls

To determine the level of various PUFAs in AMD cases and healthy controls, a detailed analysis of all the PUFAs contents was conducted among different groups. Table 2 presents plasma concentrations of omega-3 and omega-6 PUFAs in individuals with early/late-stage AMD and the healthy group. The plasma concentrations of EPA and DHA were found to be significantly lower in the early-stage AMD group compared to the healthy controls, with values of 0.69 ± 0.28 (*p* < 0.001) and 1.06 ± 0.36 (*p* = 0.003) respectively. Late-stage AMD patients exhibited lower levels of alpha linolenic acid (ALA) (*p* = 0.044) and EPA (*p* < 0.001) compared to the controls, while no significant differences were observed in other omega-3 and omega-6 PUFAs.

**Table 2:** The concentrations of omega-3 and omega-6 PUFAs in plasma in early/late-stage AMD cases and healthy controls.

**Category**	**Characteristics**	**Healthy controls** **(*n =* 105)**	**Early-stage AMD cases (*n =* 105)**	**Late-stage AMD cases** **(*n =* 105)**	***p*-value^a^**	***p*-value^b^**
	ALA (C18:3ω3)	1.13 ± 0.42	1.08 ± 0.45	1.00 ± 0.46	0.472	0.044
Omega-3	EPA (C20:5ω3)	1.00 ± 0.23	0.69 ± 0.28	0.46 ± 0.19	<0.001	<0.001
	(% of Fatty Acids)	DHA (C22:6ω3)	1.21 ± 0.38	1.06 ± 0.36	1.17 ± 0.37	0.003	0.334
	DPA (C22:5ω3)	0.65 ± 0.23	0.59 ± 0.24	0.60 ± 0.34	0.074	0.271
	AA (C20:4ω6)	7.86 ± 2.76	8.15 ± 2.24	8.67 ± 3.58	0.395	0.071
Omega-6	DGLA (C20:3ω6)	2.49 ± 0.54	2.61 ± 0.58	2.59 ± 0.60	0.113	0.183
		ADA (C22:4ω6)	0.29 ± 0.15	0.31 ± 0.16	0.32 ± 0.14	0.262	0.101
	(% of Fatty Acids)	GLA (C18:3ω6)	1.13 ± 0.48	1.18 ± 0.54	1.10 ± 0.48	0.441	0.757
		DPA (C22:5ω6)	0.21 ± 0.06	0.22 ± 0.07	0.23 ± 0.08	0.197	0.053
	LA (C18:2ω6)	34.77 ± 8.86	35.77 ± 10.49	37.12 ± 10.80	0.458	0.076

^a^Mean healthy controls vs. early-stage AMD cases; ^b^mean healthy controls vs. late-stage AMD cases. *p-*value <0.05 indicating statistical significance. ALA, alpha-linolenic acid; EPA, eicosapentaenoic acid; DHA, docosahexaenoic acid; DPA, docosapentaenoic acid; AA, arachidonic acid; DGLA, dihomo-gamma-linolenic acid; ADA, adrenic acid; GLA, gamma-linolenic acid; LA, linoleic acid.

### The correlations between PUFAs concentrations and baseline information, laboratory examinations, and ophthalmological parameters

**Figure 3:**
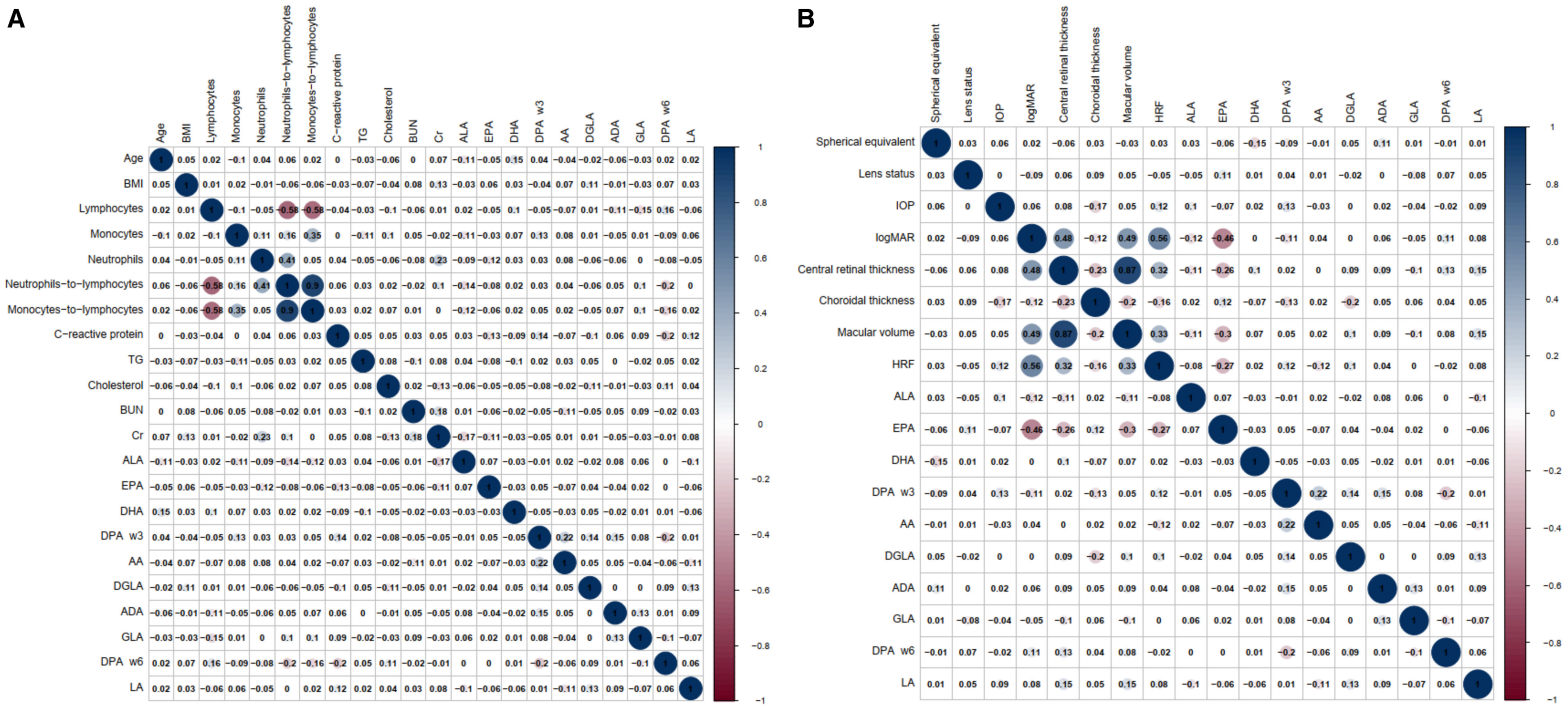
The correlation coefficient matrix, which illustrates the correlation between baseline information, blood tests results, ophthalmological characteristics, and plasma concentrations of PUFAs separately. **(A)** The correlations between baseline information, blood tests results and plasma concentrations of PUFAs. **(B)** The correlations between ophthalmological characteristics and plasma concentrations of PUFAs.

Figure 3 shows the correlation coefficient matrix and Figure 3A displays the significant correlations between baseline information, blood tests results, and plasma concentrations of PUFAs. Figure 3B highlights the correlations between plasma concentrations of PUFAs and ophthalmological characteristics. Remarkably, the study found significant negative correlations (*p* < 0.001) between EPA and logMAR, central retinal thickness, macular volume and HRF, with correlation coefficients of −0.46, −0.26, −0.3, and −0.27 respectively. These findings suggested that EPA was associated inversely with logMAR, central retinal thickness, macular volume and HRF. Additionally, HRF was another variable that our study was interested in. The correlation coefficients between HRF and logMAR, central retinal thickness, choroidal thickness and macular volume were 0.56, 0.32, −0.16, and 0.33 respectively, and were all significantly correlative (respectively, *p* < 0.001, *p* < 0.001, *p* = 0.021, and *p* < 0.001).

### Independent influencing factors for BCVA in early-stage and late-stage AMD cases

Our research also conducted multiple linear regression analyses to examine the independent influencing factors for BCVA in patients with both early-stage and late-stage AMD. Following single-factor correlation analysis and expert knowledge assessment, the relevant variables were incorporated into the multiple linear regression model, with logMAR serving as the dependent variable. The VIF values of the four independent variables in the two models were all less than 5, suggesting that there was no multicollinearity between the independent variables. The findings revealed that macular volume (*p* < 0.001, 95% confidence interval [CI]: 0.044–0.146), HRF (*p* < 0.001, 95%CI: 0.011–0.021) and EPA (*p* < 0.001, 95%CI: −0.455–−0.241) were significant influencing factors of logMAR in early-stage AMD patients. Among them, only EPA exhibited an inverse impact on logMAR (Table 3). For individuals with late-stage AMD, logMAR was significantly influenced by central retina thickness (*p* = 0.001, 95%CI: 0.0002–0.001) and HRF (*p* = 0.006, 95%CI: 0.001–0.007) (Table 4). It was noteworthy that the results of multiple linear regression analysis in the early-stage AMD group revealed a negative coefficient estimate for central retinal thickness, contradicting the sign of its correlation coefficient in the single-factor correlation analysis. Similarly, in the late-stage AMD group, the coefficient estimate of EPA was positive, contradicting the sign of its correlation coefficient in the single-factor correlation analysis.

**Table 3:** The multiple linear regression for the independent influencing factors for BCVA in early-stage AMD cases.

**Dependent variable**	**Regression coefficient**	***p*-value**	**95% confidence interval**
Central retinal thickness	−0.002	0.004	−0.004, −0.001
Macular volume	0.095	<0.001	0.044, 0.146
HRF	0.016	<0.001	0.011, 0.021
EPA	−0.348	<0.001	−0.455, −0.241

HRF, hyperreflective foci; EPA, eicosapentaenoic acid. *p*-value <0.05 indicating statistical significance.

**Table 4:** The multiple linear regression for the independent influencing factors for BCVA in late-stage AMD cases.

**Dependent variable**	**Regression coefficient**	***p*-value**	**95% confidence interval**
Central retinal thickness	0.001	0.001	0.0002, 0.001
Macular volume	−0.001	0.937	−0.029, 0.027
HRF	0.004	0.006	0.001, 0.007
EPA	0.142	0.044	0.004, 0.281

HRF, hyperreflective foci; EPA, eicosapentaenoic acid. *p*-value <0.05 indicating statistical significance.

### Comparison between good response group and poor response group

As shown in Table 5, individuals with poor responses had significantly lower baseline logMAR (*p* < 0.001), central retinal thickness (*p* = 0.002), macular volume (*p* = 0.027), and HRF (*p* = 0.024) compared to those with good responses in the late-stage AMD cases. Apart from that, it was also notable that plasma EPA concentrations in poor responders were significantly lower than those in good responders (*p* < 0.001). There was no significant difference between the two groups with respect to baseline information, blood results and other ophthalmological characteristics or plasma PUFAs concentrations.

**Table 5:** Socio-demographic, life style, blood tests and ophthalmological characteristics in good responders and poor responders after anti-VEGF treatment in the late-stage AMD cases.

**Category**	**Characteristics**	**Good responders**	**Poor responders**	***p*-value**
Socio-demography	Age (years)	71.09 ± 7.41	71.06 ± 8.47	0.985
		Gender (male/female)	33/29	23/20	0.979
Life styles	Smoking history (ever/never)	24/38	21/22	0.307
		Alcohol drinking (ever/never)	13/49	7/36	0.552
		BMI	24.44 ± 2.85	23.28 ± 3.09	0.054
		Physical activity			0.170
		None or casual	43	23		
		Frequent moderate	17	19		
		Frequent heavy	2	1		
Medical histories	Hypertension (Yes/No)	22/40	17/26	0.6676
		Diabetes (Yes/No)	16/46	17/26	0.139
		Cardiovascular diseases (Yes/No)	15/47	8/35	0.501
		Hyperlipidemia (Yes/No)	20/42	7/36	0.066
Blood tests	Lymphocytes (10^9^/mL)	1.68 ± 0.50	1.65 ± 0.56	0.782
		Monocytes (10^9^/mL)	0.40 ± 0.10	0.40 ± 0.11	0.926
		Neutrophils (10^9^/mL)	4.01 ± 1.70	4.46 ± 1.37	0.159
		Neutrophils-to-lymphocytes	2.72 ± 1.87	3.58 ± 4.39	0.173
		Monocytes-to-lymphocytes	0.27 ± 0.15	0.332 ± 0.49	0.396
		CRP (mg/L)	2.30 ± 1.40	2.30 ± 1.50	0.984
		TG (mg/d)	138.84 ± 52.16	158.02 ± 52.55	0.070
		Cholesterol (mg/dL)	191.35 ± 32.92	198.79 ± 31.37	0.253
	BUN (mg/dL)	16.24 ± 2.56	15.62 ± 2.03	0.193
	Cr (mg/dL)	0.86 ± 0.15	0.87 ± 0.13	0.603
	Spherical equivalent (D)	−0.61 ± 1.05	−0.48 ± 1.15	0.547
Ophthalmological	IOP (mmHg)	16.34 ± 3.44	17.33 ±3.18	0.143
		logMAR	0.56 ± 0.18	0.43 ± 0.10	<0.001
	characteristics	Central retinal thickness (μm)	430.52 ± 126.52	354.47 ± 1,105.09	0.002
		Choroidal thickness (μm)	143.29 ± 40.85	152.02 ± 43.74	0.302
	Macular volume (mm^3^)	13.63 ± 1.58	12.89 ± 1.72	0.027
	HRF	16.03 ± 9.15	11.98 ± 8.32	0.024
Omega-3	ALA (C18:3ω3)	0.96 ± 0.46	1.07 ± 0.44	0.236
		EPA (C20:5ω3)	0.53 ± 0.17	0.36 ± 0.18	<0.001
		DHA (C22:6ω3)	1.13 ± 0.35	1.22 ± 0.38	0.196
		DPA (C22:5ω3)	0.62 ± 0.38	0.58 ± 0.25	0.481
Omega-6	AA (C20:4ω6)	8.62 ± 3.67	8.76 ± 3.45	0.845
		DGLA (C20:3ω6)	2.69 ± 0.66	2.45 ± 0.46	0.049
		ADA (C22:4ω6)	0.34 ± 0.15	0.29 ± 0.10	0.060
		GLA (C18:3ω6)	1.10 ± 0.50	1.11 ± 0.43	0.847
		DPA (C22:5ω6)	0.23 ± 0.08	0.22 ± 0.09	0.383
	LA (C18:2ω6)	35.88 ± 10.70	38.91 ± 10.71	0.161

BMI, body mass index; CRP, C-reactive protein; TG, triglyceride; BUN, blood urea nitrogen; Cr, Creatinine; D, diopter; IOP, intraocular pressure; logMAR, logarithm of the minimum angle of resolution; HRF, hyperreflective foci; ALA, alpha-linolenic acid; EPA, eicosapentaenoic acid; DHA, docosahexaenoic acid; DPA, docosapentaenoic acid; AA, arachidonic acid; DGLA, dihomo-gamma-linolenic acid; ADA, adrenic acid; GLA, gamma-linolenic acid; LA, linoleic acid. *p*-value <0.05 indicating statistical significance.

### A prediction model for predicting the efficacy of anti-VEGF

**Figure 4:**
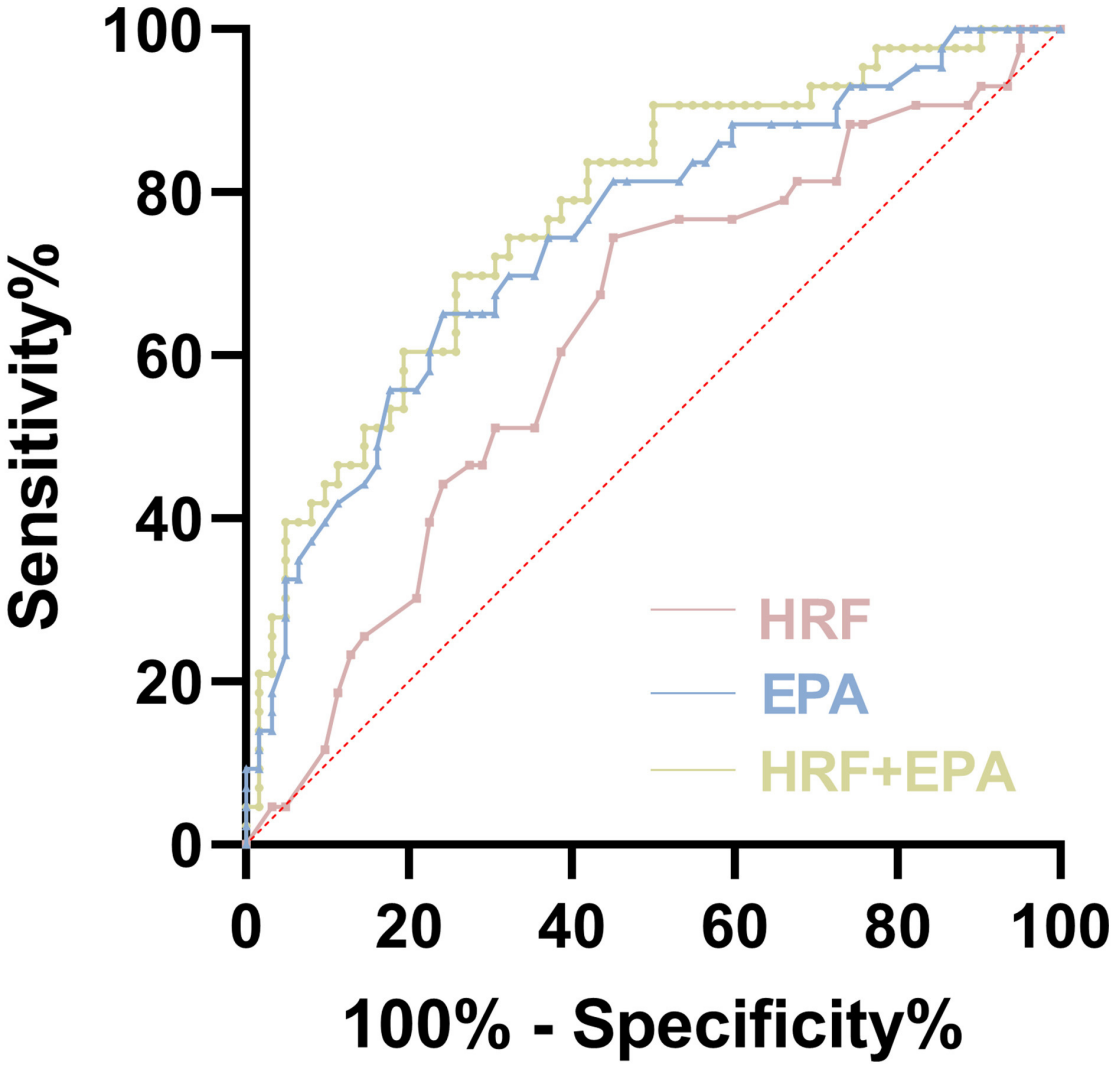
Receiver operating characteristic (ROC) curves of evaluation of HRF and plasma EPA as predictive markers of efficacy in late-stage AMD.

In order to further investigate the effect of HRF and EPA on the prediction of anti-VEGF response, we conducted an analysis using the ROC curve to compare the predictive value of HRF and EPA individually, as well as their combined effect (Figure 4). Among all the ROC curves, the combination of HRF and EPA exhibited the highest similarity to the diagnostic results provided by the retinal specialist, which is considered the reference standard. The optimal cutoff value of this ROC curve was determined using the Youden index, which maximized the sum of sensitivity (69.77%) and specificity (74.19%). This combination yielded an area under the curve (AUC) value of 0.775.

## Discussion

Currently, PUFAs contents have been regarded as potential biomarkers of various disorders, however, the role of PUFAs in AMD incidence or ophthalmological characteristics remains unclear. Besides, numerous studies try to find the factors that clinicians can utilize to forecast the response to anti-VEGF treatment through the observation and analysis of genetic variances and retinal attributes in patients with AMD. However, a consensus has yet to be reached. In this investigation, we conducted a comparison of OCT images and plasma PUFAs concentrations among early-stage AMD patients, late-stage AMD patients, and elderly control subjects devoid of any fundus diseases, the findings of this study indicate that patients with AMD exhibit lower plasma concentrations of EPA, DHA, and ALA compared to normal elderly individuals, and HRF were observed in various retinal layers of AMD patients, particularly those with late-stage AMD. Furthermore, our findings indicate a significant correlation between the visual prognosis of late-stage AMD patients and the quantity of HRF as well as the concentrations of EPA and ALA in plasma. Subsequently, the study categorized late-stage AMD patients into different response groups based on visual function and retinal structure after receiving standardized treatment. By employing ROC analysis, the study identified HRF and EPA as potential biomarkers for predicting the efficacy of anti-VEGF treatment for late-stage AMD.

Unsaturated fatty acids, particularly omega-3 PUFAs and omega-6 PUFAs, hold significant physiological importance and have garnered considerable attention from researchers (Querques et al., 2011). These two types of PUFAs, as competing substrates for identical metabolic enzymes, exhibit metabolic competitive inhibition. Omega-3 PUFAs and omega-6 PUFAs interact in a reciprocal manner to maintain bodily homeostasis. Generally, omega-3 PUFAs, including DHA, ALA, and EPA, confer beneficial effects, while omega-6 PUFAs tend to have detrimental effects (Hamsanathan et al., 2022). In our investigation, we observed significantly lower plasma concentrations of EPA and DHA in the early-stage AMD group compared to the healthy controls. Additionally, late-stage AMD patients exhibited lower levels of ALA and EPA compared to controls, with no significant differences observed in omega-6 PUFAs.

Omega-3 PUFAs play a crucial role in human physiology. Omega-3 is an indispensable nutrient for the human body and a crucial component of cell membranes. In contrast to omega-6 PUFAs and omega-9 fatty acid oleic acid, omega-3 PUFAs have the potential to enhance the elimination of protein aggregates. Omega-3 PUFAs have a direct association with the retina and can impact the cellular and intracellular functions of organ systems, as well as mitigate and prevent various eye diseases, including AMD, xerophthalmia, glaucoma, and retinitis pigmentosa. Among omega-3 PUFAs, EPA plays a significant role in the prevention of atherosclerosis, dementia, rheumatoid arthritis and neurodegenerative disorders, such as Alzheimer's disease (Mohanty et al., 2016; Hadley et al., 2016). Furthermore, several studies have demonstrated that EPA possesses the ability to enhance corneal transparency through its regulation of the inflammatory mechanism, thereby promoting the stability and functionality of the ocular surface. DHA, being a crucial constituent of various cell types, particularly retinal pigment epithelium cells, has been extensively investigated in relation to AMD. Additionally, several studies (Wong et al., 2016) have indicated that DHA is primarily present in the phospholipids of the outer segment of the photoreceptor in the eye, and it plays a crucial role in the regeneration of rhodopsin. Notably, the interaction between DHA and rhodopsin takes place subsequent to the transportation of the early Golgi balloon to the rod outer segments. Furthermore, DHA influences various aspects of photoreceptor function, including the photoreceptor membrane, neurotransmitter activity during signal transduction, rhodopsin activation, rod and cone development, nerve dendrite connectivity, and the functional maturation of the central nervous system. Furthermore, Johansson et al. (2015) discovered that physiologically significant doses of DHA in human retinal pigment epithelial cells elicit a temporary elevation in levels of reactive oxygen species (ROS) within the cells (Price et al., 2011). Additionally, pre-treatment with DHA effectively rescues the cells from cell cycle arrest caused by misfolded proteins or oxidative stress. The initial stage of AMD is linked to the accumulation of intracellular lipofuscin and extracellular deposits. DHA has the ability to stimulate the production of endogenous antioxidants and facilitate selective autophagy of misfolded proteins. These findings suggest that omega-3 PUFAs may potentially mitigate the risk of developing diseases associated with protein aggregates such as AMD. Furthermore, the potential mechanisms by which omega-3 PUFAs regulate AMD encompass the reduction of cytokines, inhibition of cell proliferation, and modulation of antigen presentation, among others. However, a comprehensive understanding of the specific mechanism necessitates further investigation. In summary, both omega-3 and omega-6 PUFAs can contribute to the pathogenesis and progression of AMD as local hormones involved in inflammatory processes. Notably, omega-3 PUFAs, such as EPA, ALA, and DHA, possess anti-inflammatory properties, which enable them to modulate immune and inflammatory cell function, thereby exerting a therapeutic and preventive role in AMD.

In our study, we observed a notable decrease in plasma concentrations of EPA not only in individuals with early-stage AMD, but also in those with late-stage AMD, when compared to the healthy controls. These findings imply that EPA may have a significant involvement in the development of all AMD stages. However, the precise association between AMD pathogenesis and EPA deficiency has not been fully established, and the specific preventive and protective mechanisms of EPA against AMD remain uncertain. Consequently, we selected EPA as the primary focus for subsequent investigations.

In our investigation, we observed a negative association between EPA levels and central retinal thickness as well as macular volume, and a positive correlation between plasma EPA concentration and vision. This indicates a potential involvement of EPA deficiency in the progression of AMD, as well as its association with vision impairment and retinal structure damage. Prior research has demonstrated that EPA metabolism generates anti-inflammatory lipid mediators in healthy cells (Barabino et al., 2017; Yanai et al., 2014). Moreover, EPA has been found to decrease the size of retinal vessels by promoting vascular regeneration following injury, thereby mitigating hypoxia-induced neovascularization and safeguarding the proper functioning of the retina (Yanai et al., 2014). Our findings provide further support for this conclusion. Besides, we elucidated the previously unexplored relationship between EPA and retinal HRF, and we found that EPA was associated inversely with HRF in AMD patients. Afterwards, we analyzed the influence factors of BCVA in patients with early and late-stage AMD. Results demonstrated that BCVA was significantly influenced by macular volume, HRF, and EPA in early-stage AMD patients and only EPA showed reversibility. In individuals with late-stage AMD, both central retinal thickness and HRF affected BCVA significantly. Remarkably, the results of multiple linear regression of central retinal thickness and EPA in their respective groups were contrary to the results of single- factor analysis. This might suggest that in the early-stage AMD group, there was a correlation between central retinal thickness and BCVA, but no significant impact on BCVA. Additionally, EPA levels were found to be similar in the late-stage AMD group.

The HRF was another variable we examined in our study. Our findings indicated a notable presence of HRF on OCTA images among patients diagnosed with AMD, particularly those in advanced stages, aligning with previous research outcomes. Leuschen et al. (2013) found that these dot-shaped intra-retinal lesions are present in approximately 50 per cent of AMD eyes among 313 patients. Another study also demonstrated that the distribution of intraretinal HRF in the macula increased in quantity and migrated from the outer to the inner retinal layers during 2 years of disease progression (Christenbury et al., 2013; Damian and Nicoara, 2022). It is worth noting that HRF can be detected on OCT scans in patients with various retinal conditions, including AMD, DME, Stargardt disease, and retinitis pigmentosa.

Histological investigations (Zanzottera et al., 2015; Pang et al., 2015; Chen et al., 2016) revealed that RPE cells exhibiting forward migration (above the outer plexiform layer) exhibited HRF on OCT imaging. Furthermore, Zanzottera et al. (2015) highlighted that the HRF observed on OCT scans of AMD eyes may comprise both RPE and lipid-rich microglia, indicating that the retinal HRF originates from at least two distinct cellular sources. Other studies showed that HRF activated migrated RPE cells in the internal retinal layers, induced by cytokines and other inflammatory mediators as a response to complement activation (Pieroni et al., 2006; Mitsuhiro et al., 2003). During follow up, researchers found that HRF progressively proliferated and migrated toward the internal retinal layers (Folgar et al., 2016). Therefore, HRF might reflect a dynamic process of retinal inflammation in AMD.

To date, the origin of the retinal HRF remains a subject of debate, with numerous hypotheses proposed. However, it is widely acknowledged among researchers that the HRF serves as a biomarker for inflammatory response, and the activation and aggregation of microglia may be attributed to the presence of HRF (Vujosevic et al., 2017; Fragiotta et al., 2021). Furthermore, during the early stages of DR, HRF were primarily located in the inner layer of the retina, while in the middle and late stages of DR, they were predominantly found in the outer layer of the retina, aligning with the migration pathway of activated microglia (Ebneter et al., 2017), which is consistent with the results we have observed in OCT images of AMD patients. Under normal physiological circumstances, the microglia distribute in the inner layer of the retina and serve to monitor changes in the retinal microenvironment. Upon exposure to inflammatory mediators, these microglia become activated and migrate toward the outer retina, thereby perpetuating inflammation and leading to damage of nerve cells. Furthermore, the presence of HRF has been recognized as a biomarker for various retinal diseases. However, its impact on disease prognosis varies across different studies. For instance, Yoshitake et al. (2020) discovered that DME patients with retinal HRF exhibited a more favorable response to treatment, as evidenced by higher visual acuity improvement and a more substantial reduction in central retinal thickness. Conversely, numerous studies have reported an association between high reflex points and a poorer visual prognosis (Uji et al., 2012; Nishijima et al., 2014; Kang et al., 2016). In our investigation, we observed a positive correlation between the quantity of HRF and central retinal thickness, macular volume, and logMAR in AMD patients. It is hypothesized that HRF has the potential to serve as a biomarker for predicting the effectiveness of treatment for nAMD. The presence of HRF indicates the potential need for alternative therapeutic targets. It is possible that nAMD patients with HRF may exhibit a more favorable response to hormone sustained-release drugs compared to single anti-VEGF therapy. Additionally, PUFAs play a crucial role in the composition of phospholipid bilayers within cell membranes, including those of microglia. Abnormal concentrations of PUFAs, particularly a decrease in omega-3 PUFAs, may contribute to the activation of microglia. Additionally, in the pathogenesis of AMD, microglia are believed to have a significant impact on the inflammatory response. As previously mentioned, HRF may be indicative of activated microglia within the retina. Hence, it is plausible to establish a connection between PUFAs and HRF, and our study also revealed noteworthy inverse associations between EPA and HRF. However, it is crucial to note that PUFAs alone cannot be deemed as the sole determinant of HRF. In this particular investigation, we employed EPA in conjunction with HRF as an indicator to prognosticate the likelihood of favorable outcomes in late-stage AMD patients undergoing anti-VEGF therapy. The findings demonstrated that the amalgamation of EPA and HRF exhibited superior diagnostic efficacy compared to their individual utilization.

The implementation of anti-VEGF therapy has demonstrated a noteworthy reduction in severe visual impairment among individuals diagnosed with neovascular AMD. Nevertheless, the extent of benefit experienced by patients varies considerably. This heterogeneity in response can be ascribed to considerable inter-individual variations in characteristic traits. Consequently, the identification of biomarkers capable of assessing and forecasting the effectiveness of anti-VEGF treatment assumes paramount importance, carrying significant clinical implications. In this study, we utilized morphological and functional outcomes to assess the therapeutic response in late-stage AMD patients. And the results indicated that poor responders had significantly lower EPA and plasma levels of EPA in baseline than good responders, suggesting that late-stage AMD patients with low plasma EPA concentrations may require treatment other than anti-VEGF in the early stage. Accumulating researches (Merle et al., 2013, 2014; Seddon et al., 2003, 2006; Chong et al., 2008) revealed a negative association between the consumption of fish, a dietary source of marine omega-3, and the risk of developing AMD. The preventive impact of increased fish intake on AMD is closely linked to the presence of marine omega-3. Additionally, certain studies have indicated a correlation between omega-3 deficiency in photoreceptors and the occurrence of AMD (Serini et al., 2011). Our study indicated a potential protective effect of omega-3 PUFAs, specifically EPA and DHA, in the development of AMD. This also demonstrated the beneficial impact of dietary supplementation with omega-3 PUFAs on maintaining ocular health and preventing AMD. Omega-3 PUFAs might be used as an adjunct treatment option for late-stage AMD to reduce the frequency of anti-VEGF therapy as well. Notably, our study pioneers the development of a curative effect prediction model that utilizes circulating blood PUFAs and retinal HRF to determine the effectiveness of anti-VEGF treatment in AMD patients. This model can aid healthcare professionals in identifying patients who may not respond favorably to single anti-VEGF therapy during the early-stage AMD.

However, it is important to acknowledge the limitations of our study. Firstly, it is crucial to recognize that AMD is a chronic disease, and the effectiveness of late-stage AMD treatment targeting VEGF may exhibit recurrent fluctuations. Therefore, longer-term follow-up studies are needed to assess the stability of the biomarkers identified. Secondly, the precise role and mechanism of EPA in AMD pathogenesis remain unexplored; our team is currently pursuing fundamental research in this area. Moreover, although dietary habits, which can affect plasma PUFA levels, were considered, they were not comprehensively controlled in this study. Future research should include detailed dietary assessments to better account for this variable. Lastly, being a single-center study, our findings may not be generalizable to broader populations. Larger-scale multicenter studies are necessary to validate the prognostic significance of EPA and HRF in AMD patients and to clarify the underlying mechanisms of their interactions. These efforts are crucial for improving early identification of patients who may not respond optimally to anti-VEGF therapy and for developing personalized treatment strategies. However, our study provides additional evidence to support the potential involvement of EPA in the prevention of late-stage AMD. Furthermore, our findings establish a foundation for future investigations into the efficacy of oral EPA supplements in preventing and slowing the progression of AMD. Additionally, we have elucidated the previously unexplored relationship between EPA concentrations and retinal HRF. Notably, our study has also identified the significant predictive value of plasma EPA and HRF in determining the response to anti-VEGF treatment.

## Conclusion

In summary, the findings of this study indicate that EPA concentration was lower in both early-stage and late-stage AMD cases and it was found that lower EPA concentration was related to poor BCVA. Furthermore, the combination of baseline HRF and plasma EPA concentration can serve as a reliable biomarker for predicting the effectiveness of late-stage AMD treatment. However, further investigations involving extended observation periods and larger cohorts are necessary to validate the enduring stability of the biomarkers identified in this investigation.

## Data Availability

The original contributions presented in the study are included in the article/supplementary material, further inquiries can be directed to the corresponding authors.
